# Clinical effectiveness of fecal microbial transplantation for metabolic syndrome: Advances in clinical efficacy and multi-omics research

**DOI:** 10.1016/j.crmicr.2025.100415

**Published:** 2025-06-05

**Authors:** Hanrui Wang, Jiaxing Tian, Jia Mi

**Affiliations:** a College of Traditional Chinese Medicine, Changchun University of Chinese Medicine, Changchun 130117, China; b Institute of Metabolic Diseases, Guang'anmen Hospital, China Academy of Chinese Medical Sciences, Beijing 100053, China; c Department of Endocrinology, The First Affiliated Hospital of Changchun University of Chinese Medicine, Changchun 130021, China

**Keywords:** Metabolic syndrome (MetS), Fecal microbial transplantation (FMT), Gut microbes, Obesity, Insulin sensitivity, Multi-omics

## Abstract

Even though metabolic syndrome (MetS) poses a serious risk to human health and life, existing treatment approaches are not very effective. The impact of gut bacteria on host metabolism has been the subject of numerous research, and fecal microbial transplantation (FMT) has demonstrated great promise in reducing insulin resistance and abdominal obesity in individuals with metabolic syndrome. These FMT investigations have connected alterations in the gut microbiota to clinical indicators of insulin resistance and obesity, in addition to using high-throughput methods to analyze the gut microbiome, metabolome, and epigenome of peripheral blood mononuclear cells in patients with MetS. It is still necessary to clarify and assess the clinical effectiveness and mode of action of FMT in the management of MetS. This review examines the connection between gut bacteria and MetS, the effectiveness of FMT as a treatment, and the changes in the gut microbiome, metabolome, epigenome, and other histones following the intervention. We also discuss the safety of FMT and suggest areas for further investigation.

## Highlights

• Fecal microbial transplantation has demonstrated promise in reducing insulin resistance and abdominal obesity.
• The predominant taxa in the individual donors or the total samples were Bacteroidetes and Firmicutes.
• Changes in metabolite levels measured in feces and plasma after fecal microbial transplantation intervention were focused on bile acids, short-chain fatty acids, amino acids, and some small-molecule lipids.
• In host peripheral blood mononuclear cells, there was a significant change in the DNA methylation of the Actin filament-associated protein 1 gene.

## Graphical abstract

**Figure:**
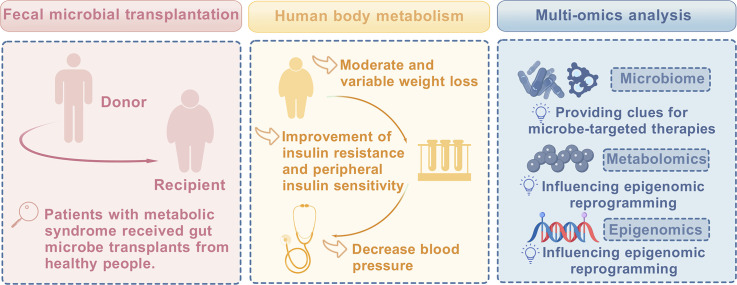

## 1 Introduction

Metabolic syndrome (MetS) is one of the most significant health problems in the world today (Cefalu et al., 2015). If treatment is not received, it can result in other metabolic disorders or diseases such as type 2 diabetes mellitus (T2DM), steatotic liver disease associated with metabolic dysfunction, cardiovascular disease (CVD), and chronic kidney disease (CKD) (Ndumele et al., 2023). Clinical manifestations of MetS include abdominal obesity, insulin resistance, hypertension, high triglycerides, low high-density lipoprotein (HDL), and systemic inflammation (Neeland et al., 2024). Although the underlying pathophysiological mechanisms of MetS are not fully understood, the Human Microbiome Project and the development of multi-omics investigations have revealed the relationship between gut microbiome and human disease. Disturbances in the gut microbiota have also been found to be a risk factor for the development of MetS, and alterations in the gut microbiome have been connected to insulin sensitivity, glucose metabolism, and obesity (Nicholson et al., 2012; Utzschneider et al., 2016). Additionally, fecal microbial transplantation (FMT) has gained attention as a therapeutic approach that targets the gut microbiota and has been tried to treat several illnesses (van Prehn et al., 2021; D'Haens and Jobin, 2019).

FMT, a therapeutic strategy that effectively treats recurrent *Clostridioides difficile* infection (CDI), targets the gut microbiome. According to clinical guidelines and consensus, recurrent or refractory CDI can be treated with microbiome transplantation (van Prehn et al., 2021). In addition to this, based on the research on microbiota, FMT has been gradually extended for the treatment of gastrointestinal and other diseases outside the gastrointestinal tract, central nervous system diseases, and MetS (D'Haens and Jobin, 2019; Mullish et al., 2023). Several studies suggest that FMT, as a unique therapeutic approach, may influence MetS by altering the composition of the gut microbiome and its metabolites.

The transplantation of donor gut microbiota may be beneficial to the recipients' (MetS sufferers') gut microbes. Through their metabolic activities, gut microbiota generates a variety of metabolites, including bile acids (BAs), short-chain fatty acids (SCFAs), and amino acids (AAs). These metabolites impact the metabolic homeostasis of the host, and consequently improve abdominal obesity and alter fat distribution to a certain extent (Leong et al., 2020; Zuppi et al., 2024). Additionally, the FMT can enhance glucose metabolism and insulin sensitivity (Mocanu et al., 2021; Zhang et al., 2024; Kootte et al., 2017). However, in addition to the microbial diversity and composition of donor feces, the effectiveness of FMT is strongly related to the initial gut microbial composition and diversity, fiber and nutrient intake, dietary behaviors, and microenvironmental characteristics of the recipient (Zhang et al., 2024).

The number of clinical investigations into FMT for the treatment of MetS has recently increased, allowing us to gain more insights into the treatment's clinical effectiveness, as well as its potential microbial targets and mechanisms of action (Leong et al., 2020; Zuppi et al., 2024; Mocanu et al., 2021; Zhang et al., 2024; Kootte et al., 2017). At this point, we would like to provide an overview of the research methodology used in these clinical trials. To start our analysis, we will explore the relationship between gut microbiota and MetS. Subsequently, we will discuss how FMT can modify the microbial ecology of individuals with MetS, thereby enhancing their clinical metabolism. Additionally, we will investigate the effects of FMT intervention on the metabolome, epigenome, gut microbiota, and other multi-omics in individuals with MetS. Research on FMT provides new ideas for the treatment of MetS and offer new perspectives and clues for microbe-targeted treatment of MetS.

## 2 The connection between gut microbiota and metabolic syndrome

The microbiota is the entire group of microbes that reside in a certain location. This encompasses not only bacteria but also viruses, fungi, protozoa, and archaea (Hou et al., 2022). Of these, the gut microbiome is the most diverse in the human body (Turnbaugh et al., 2007). Throughout the long-term co-evolution process, the microbiota in the human body has gradually established a symbiotic relationship with the host. Among its many functions will be the regulation of the host's gene expression, the function of the intestinal barrier, the metabolism of nutrients, and the overall immune system (Liu et al., 2017). Although bacteria are typically the subject of FMT studies, intestinal phages' function in FMT has also been examined, as have fecal filtrate transplants (FFT) (Ott et al., 2017) and fecal virosome transplants (FVT) (Lin et al., 2019; Draper et al., 2020). The bulk of the gut microbiota in a healthy state is composed of Firmicutes, Bacteroidetes, Proteobacteria, and Actinomycetes. There are other phyla in the gut as well, such as Clostridium and Verrucomicrobia (Jandhyala et al., 2015).

**Fig. 1:**
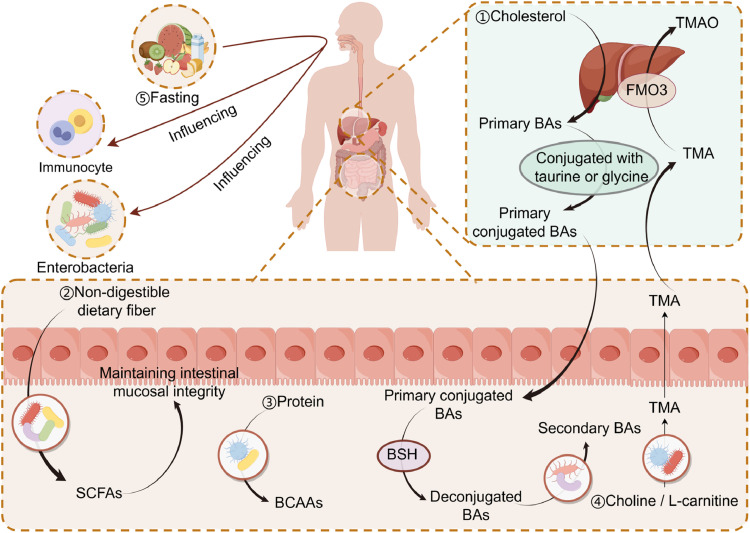
An illustration of how gut microbiota contribute to metabolic syndrome. (i) The liver transforms cholesterol into primary BAs. Primary BAs are mainly bound to glycine or taurine before being carried to the gallbladder as bile and stored there. Primary conjugated BAs are released into the intestinal lumen when dietary fat is consumed. Primary conjugated BAs are broken down by BSH-producing bacteria, and they are then further modified into secondary BAs by other microbiome members. (ii) Intestinal microbes digest dietary fiber in the gut to create SCFAs. (iii) The gut bacteria can use and break down proteins to create BCAAs. (iv) Choline and L-carnitine combine to create TMA in the presence of gut bacteria. TMA is absorbed and then transported to the liver, where FMO3 oxidizes it to form TMAO. (v) Fasting affects the gut microbiota and immune system. BAs, bile acids; BSH, Bile salt hydrolase; SCFAs, short chain fatty acids; BCAAs, branch chain amino acids; TMA, trimethylamine; FMO3, flavin monooxygenase 3; TMAO, Trimethylamine-N-oxide. By Figdraw.

Gut microbiota impacts human metabolism through a variety of pathways, such as metabolite production, energy metabolism, immunological regulation, and inflammatory reactions (Dabke et al., 2019). Metabolites from the microbiota, specifically branched-chain amino acids (BCAAs), trimethylamine N-oxide (TMAO), tryptophan, indole derivatives, and BAs and SCFAs, have been implicated in the pathogenesis of metabolic diseases (Agus et al., 2021). Gut microbiota can catabolize certain nutrients, which leads to the creation of a wide range of metabolites. BAs controlled by gut microbiota affect insulin sensitivity, insulin secretion, glycogen, and lipid synthesis (Collins et al., 2023). SCFAs, which are metabolic byproducts of microbial fermentation of dietary fibers, can decrease low-grade inflammation, enhance dyslipidemia, inhibit intracellular lipolysis, and improve intestinal barrier integrity. Additionally, they actively support better insulin sensitivity, glucose homeostasis, and weight control (Canfora et al., 2015). The gut microbiota generates and uses BCAAs, which are crucial for maintaining homeostasis, via regulating protein synthesis, glucose and lipid metabolism, insulin resistance (Solon-Biet et al., 2019). Toxic substances including TMAO, choline, and L-carnitine then reach the circulation due to abnormal gut microbial metabolism, which impairs insulin sensitivity and glucose homeostasis (Agus et al., 2021). Dietary interventions can also alter the composition of the gut microbiota, thereby affecting human metabolism. Clinical research has shown that fasting affects humans' immune system and gut bacteria (Fig. 1). It also causes changes in the abundance of several core symbionts, with significant changes in the abundance of Firmicutes following fasting, however, these changes were reversed upon refeeding. At the end of the refeeding period, sustained depletion can be seen in *Enterobacteriaceae*, especially *Escherichia coli*. Monocytes and TCRγ/δ T cells increased, whereas CD3, CD4 T cells, and CD19 B cells decreased after simultaneous fasting (Maifeld et al., 2021).

In general, gut microbiota are closely related to the metabolic activities of the human body (Dabke et al., 2019). Metabolites generated by the gut microbiota have a major impact on the physiopathology of MetS (Agus et al., 2021). Microbial metabolites, particularly BAs, SCFAs, BCAAs, and TMAO, can affect human metabolism and may be used as biomarkers for illness prognosis and early detection (Agus et al., 2021; Collins et al., 2023; Canfora et al., 2015; Solon-Biet et al., 2019). FMT, on the other hand, involves the transplantation of microbiota to alter the host's gut microbial species and proportions, as well as their derivatives, further altering the host's intestinal barrier, insulin sensitivity, and glucolipid metabolism.

## 3 Impact of fecal microbial transplantation on human metabolism

### 3.1 Impact of fecal microbial transplantation on obesity

In recent years, obesity has spread around the world, impacting people's health (Rubino et al., 2025; Garvey et al., 2016; Yumuk et al., 2015) and playing a major role in MetS (Després and Lemieux, 2006). Most conventional treatments for obesity and related disorders are somewhat ineffectual. Although lifestyle interventions and medication can lead to weight loss, they are ineffective, and compliance with long-term adherence is low (Bessesen and Van Gaal, 2018). Bariatric surgery can aid in weight loss, but there is a risk that it can result in serious complications (Coutant et al., 2017). Pioneering mice studies have shown that lean and obese phenotypes can be transferred from human donors' fecal microbiota (Ridaura et al., 2013). In prior studies, the field of microbiome research has made useful progress in determining the link between gut microbiota changes and obesity (Maruvada et al., 2017; Ley et al., 2005). The gut microbiome can indirectly participate in glucose and lipid metabolism in the body and plays an important role in regulating body weight (Leong et al., 2018). Obesity has also been associated with changes in the gut microbiota, which impacts the host's immune system and breaks down indigestible fiber to increase energy intake. By digesting these nutrients, the microbiota can ferment dietary fiber, and the products, SCFAs, increase the release of glucagon-like peptide 1 (GLP-1), as well as modulate the bile acid pathway to facilitate the digestion of fats and oils (Sanna et al., 2019). This information suggests that FMT could be used as a therapeutic strategy to target the microbiota (Hartstra et al., 2015; de Clercq et al., 2016). FMT is recognized as a potential treatment for obesity and related metabolic diseases.

An open-label pilot trial of intense FMT in obese individuals found that nine participants experienced varying degrees of weight loss at the 12-week follow-up after the intensive FMT intervention. This implies that obese patients experienced moderate and variable weight loss as a result of the intense FMT session (Zhang et al., 2022). The FMT experiments by Annefleur et al. and Valentin et al. reached the same conclusions (Koopen et al., 2021). FMT can alter the intestinal mucosal microbiome of obese patients, with more significant changes in the colonic mucosal microbiome, while the duodenal mucosal microbiome remained essentially stable. The recovery of mucosal *Bacteroides* may be associated with weight loss in obese individuals after FMT intervention (Zhang et al., 2022). Research has also preliminarily demonstrated that FMT can change the distribution of fat and reduce visceral fat in participants. Trials by Karen et al. and Brooke et al. both showed that after FMT intervention, although the BMI SDS did not undergo a significant change, the android-to-gynoid-fat (A/G) ratio was slightly reduced, which suggests that visceral obesity might have undergone a reduced, which is equivalent to reducing the proportion of abdominal fat while changing the fat distribution (Leong et al., 2020; Zuppi et al., 2024). In addition, the majority of participants in the FMT-treated group experienced remission of MetS after 26 weeks of intervention (Leong et al., 2020). Then, in a systematic evaluation and meta-analysis, FMT was found to be significantly and negatively linked with most indices of abdominal obesity, including blood pressure, cholesterol, HOMA-IR (Homeostasis Model Assessment-Insulin Resistance), fasting glucose, and caloric intake (Zecheng et al., 2023).

### 3.2 Impact of fecal microbial transplantation on insulin resistance

Insulin resistance (IR) is a pathological condition in which the target organs of insulin action are hypersensitive to insulin; in other words, a normal insulin dose delivers less than the normal biological effect (Muscogiuri et al., 2022; Petersen and Shulman, 2018). Insulin resistance is a significant pathology in MetS. Untreated or protracted insulin resistance can result in hyperglycemia, T2DM, and other diseases (Zhao et al., 2023). Insulin resistance results in impaired peripheral glucose and a failure of insulin to suppress hepatic gluconeogenesis and lipolysis (Petersen and Shulman, 2018; Samuel and Shulman, 2016). Insulin resistance can increase circulating glucose levels, a phenomenon that leads to a compensatory increase in insulin production in pancreatic β-cells, which in turn leads to hyperinsulinemia and a vicious cycle that further increases insulin resistance (Petersen and Shulman, 2018; Samuel and Shulman, 2012; Samuel and Shulman, 2016). Insulin resistance leads to diminished insulin action in the liver, muscle and adipose tissue and failure to maintain peripheral glucose homeostasis (Jang and Lee, 2021). Numerous factors can lead to insulin resistance, and studies have shown that people with T2DM have a moderate ecological dysbiosis of the gut microbiota, in which the abundance of some generalized butyrate-producing bacteria declines and the abundance of various opportunistic pathogens increases (Qin et al., 2012). The gut microbiota is recognized as one of the key endogenous factors causing insulin resistance. Changes in the gut microbiota and the composition of its derivatives are significantly associated with insulin resistance, diabetes, and host metabolic processes (Pedersen et al., 2016).

Lean donor fecal microbiota transplantation can effectively improve glucose metabolism in recipients with MetS (Leong et al., 2020; Kootte et al., 2017; de Groot et al., 2020). Altering the composition of the gut microbiota by infusing healthy lean donor feces has a (short-term) positive impact on peripheral insulin sensitivity in patients with MetS (Leong et al., 2020). Moreover, the recipient's baseline fecal microbiota characteristics, diet, and microenvironment (Zhang et al., 2024) all influenced the efficacy of FMT after the intervention; metabolic responders had lower initial fecal microbiota diversity (Zhang et al., 2024; Kootte et al., 2017). FMT combined with low fermentable fiber could better improve insulin sensitivity after FMT by differentially modulating the implantation of selected bacterial taxa and the enteroendocrine axis (restoration of physiological patterns of GLP-1 secretion) (Mocanu et al., 2021). Meanwhile, a randomized controlled trial that used allogenic FMT from MetS donor (METS-D) feces versus feces from post-Roux-en-Y gastric bypass donor (RYGB-D) feces as a control group also demonstrated that transplantation using fecal microbiota from metabolically impaired obese donors also temporarily worsened insulin sensitivity in MetS recipients. Furthermore, the study found no discernible alteration in insulin sensitivity in the post-Roux-en-Y gastric bypass receptor (RYGB-R). This might be a result of the reduced baseline glucose disappearance (Rd) rates in all RYGB-R non-responders. It also suggests that in individuals who are already poorly insulin-sensitive, insufficient residual metabolic activity and a diminished effect of microbial intervention are expected (de Groot et al., 2020)(Table 1).

**Table 1:** Clinical Trial With Fecal Microbial Transplantation for Metabolic Syndrome.

First author	Methodology	Main outcomes	References
Mocanu V	The study used gut microbiota from lean donors and performed fecal microbial transplantation (FMT) in oral capsules. Different types of fiber were also supplemented daily as an adjunct to FMT therapy, including high fermentable and low fermentable fiber supplements.	A single dose of FMT combined with daily non-fermentable fiber supplementation can successfully improve insulin resistance in patients with severe obesity and metabolic syndrome.	(Mocanu et al., 2021)
Zhang Z	FMT combined with dietary fiber supplementation, along with subgroup analysis. Participants undergoing FMT were categorized into HOMA-IR responders and HOMA-IR non-responders to further assess the effects of plasma bile acid changes, receptor parameters and gastrointestinal factors on microbiota implantation and insulin resistance.	Baseline fecal microbiota composition predicted improvement in HOMA-IR. Responders had significantly different levels of microbial implantation compared to non-responders. and correlated with subjects' gut microbial diversity, fiber and nutrient intake, inflammatory markers, and bile acid derivative levels at baseline.	(Zhang et al., 2024)
Kootte RS	This trial investigated the effects of lean donor (allogenic) versus own (autologous) fecal microbiota transplantation to male recipients with metabolic syndrome.	Modification of gut microbiota composition by infusion of healthy lean donor feces has a (short-term) beneficial effect on peripheral insulin sensitivity in patients with metabolic syndrome. The beneficial effects of lean donor FMT on glucose metabolism are associated with changes in gut microbiota and plasma metabolites and can be predicted based on baseline fecal microbiota composition.	(Kootte et al., 2017)
Koopen AM	All male subjects with metabolic syndrome adhered to the Mediterranean diet for a total of 8 weeks, with a 2-week break-in period (week −2 to week 0). After these 2 weeks, subjects were randomly assigned (using computerized randomization) to either allogeneic FMT (receiving feces from a lean healthy donor) or autologous FMT (receiving their own feces).	Consumption of the Mediterranean diet resulted in a reduction in body weight, HOMA-IR, and lipid levels. However, the synergistic beneficial metabolic effects of the Mediterranean diet in combination with lean donor FMT on glucose metabolism were not achieved.	(Koopen et al., 2021)
de Groot P	This trial investigated whether allogenic FMT using faeces from post-Roux-en-Y gastric bypass donors (RYGB-D) has a short-term effect on glucose metabolism, intestinal transit time, and adipose tissue inflammation in treatment-naïve, obese, and insulin-resistant male subjects compared with using feces from metabolic syndrome donors (METS-D).	Allogenic FMT with METS-D reduces insulin sensitivity in metabolic syndrome recipients compared to after RYGB-D use. In addition, a trend toward faster intestinal transit time after RYGB-D FMT was observed. They also observed changes in fecal bile acids (increased lithocholic, deoxycholic, and (iso)lithocholic acid after METS-D FMT), markers of inflammation, and changes in several gut microbiota taxa.	(de Groot et al., 2020)
Manrique P	In this trial, subjects were randomly assigned to the control treatment group (self-fecal microbial transplant) and treatment (healthy donor fecal microbial transplant) treatment groups. Fecal samples were collected from the subjects and bacteriophage-like particles were purified and sequenced.	In this trial, FMT from healthy donors was found to significantly alter the intestinal phage community. Subjects with better clinical outcomes were closer to the healthy donor group, suggesting that their phage community was more similar to that of the healthy donor throughout the course of treatment.	(Manrique et al., 2021)
van der Vossen EWJ	This trial was a randomized controlled trial of allogenic (lean donor) and autologous FMT. The trial examined the effects of allogeneic or autologous FMTs on the gut microbiome, plasma metabolome, and epigenomic (DNA methylation) reprogramming in peripheral blood mononuclear cells in individuals with metabolic syndrome measured at baseline and after 6 weeks.	FMT had significant effects on the gut microbiome, host plasma metabolome and epigenome of peripheral blood mononuclear cells. Experiments showed that larger gut microbiota shifts were associated with allogenic FMT than with autologous FMT. Most notably, the introduction of *Prevotella* ASVs directly correlated with methylation of AFAP1.	(van der Vossen et al., 2021)

AFAP1, Actin filament-associated protein 1; HOMA-IR, Homeostasis Model Assessment-Insulin Resistance; FMT, fecal microbial transplantation; METS-D, metabolic syndrome donor; RYGB-D, post-Roux-en-Y gastric bypass donor.

Although allogenic FMT trials show a significant improvement in peripheral insulin sensitivity overall, there are a lot of individual differences in treatment effects, and some studies have found no significant change in peripheral or hepatic insulin sensitivity following FMT (Koopen et al., 2021). For this reason, some researchers have further categorized recipients based on Rd into responders (>10 % increase in Rd) and non-responders (<10 % increase in Rd). The responding group's hepatic and peripheral insulin sensitivity significantly increased, but the non-responding group exhibited no change (Kootte et al., 2017). This condition may result from factors such as the type, abundance, and diversity of baseline microbiota present in the donor or receiver, or it may be that the diet affected FMT (Koopen et al., 2021). Nevertheless, at week 18 after FMT, peripheral or hepatic insulin sensitivity was not significantly impacted by single or repeated allogenic FMT, suggesting that FMT had no long-term effect on insulin resistance (Kootte et al., 2017). This long-term change may be explained by the host immune system adapting to the changed gut microbiota after FMT (Zeevi et al., 2015).

## 4 Multi-omics analysis of fecal microbial transplantation

### 4.1 Microbiomics

**Fig. 2:**
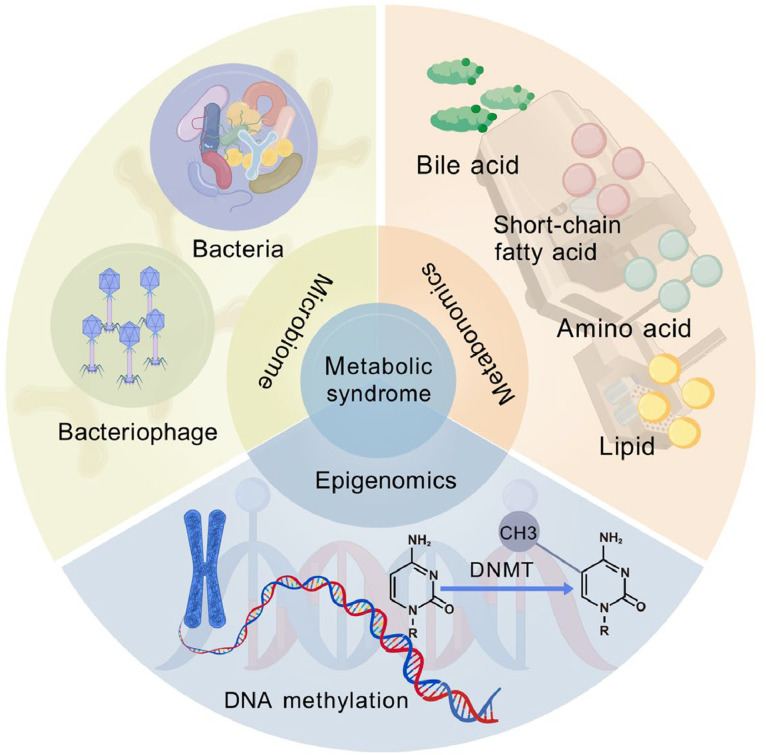
A combined analysis plot of metabolomics, epigenomics, and microbiomics. Created with BioGDP.com (Jiang et al., 2024).

The microbiome is the totality of microbiota and their genetic information in a specific ecosystem or environment. It includes interactions between microbes and other animals as well as interactions between microbes and their surroundings (Marchesi and Ravel, 2015). The gut microbiota is a group of microbes that colonize the intestinal tract, and the main subjects of study are bacteria and viruses (phages) (Hou et al., 2022). Microbiomics research methods mainly include sequencing technology and data analysis technology. The commonly adopted sequencing techniques include amplicon sequencing, macrogenome sequencing, and macrotranscriptome sequencing, which are used for microbial gene and transcript-level research (Doyle et al., 2025; Duan et al., 2025). They can provide a more comprehensive understanding of the microbial diversity, population structure, functional activity, interactions, and the relationship with the environment (Fig. 2).

#### 4.1.1 Gut bacteria

Multiple clinical investigations have demonstrated that FMT causes the recipient's gut microbiota to change toward the microbial composition of a healthy donor, alternatively, it may be characterized as a transplant of the donor's gut microbiota into the recipient. Different recipients do not consistently colonize after receiving fecal transplants from the same donor (Zhang et al., 2022), and donor microbial implantation is influenced by baseline factors of the recipient such as microbiota composition, variety, nutrition, and microenvironmental characteristics (Zhang et al., 2024). Following the FMT intervention, there was a significant shift in the microbiota composition (beta diversity) (Mocanu et al., 2021), with an increase in Shannon diversity (Leong et al., 2020; Zhang et al., 2024) and microbial abundance (Mocanu et al., 2021; Wu et al., 2023). The extent of this change was mostly determined by the subjects' diet, lipid metabolism, and baseline gut microbial diversity (Zhang et al., 2024). Additionally, FMT causes changes in the colon's and duodenum's mucosal microbiome, primarily in the *Bacteroides* genus (Zhang et al., 2022). However, a wider range of alterations in the gut bacteria seen in the feces is shown when compared to the intestinal mucosal microbiome.

After the FMT intervention, a microbiomic analysis of the microbiota in feces was carried out. From the phylum level, the predominant taxa in the individual donors or in the total samples were *Bacteroidetes* and *Firmicutes*, followed by *Proteobacteria, Actinobacteria*, and *Verrucomicrobia*, which underwent changes during FMT. At the genus level, the most significant alterations were in *Bacteroides, Alistipes, Ruminococcus*, and *Bifidobacterium*; followed by *Faecalibacterium, Clostridium, Christensenella*, and *Desulfovibrio*. The FMT study discovered that, at the species level, *Alistipes finegoldii* (Leong et al., 2020; Zhang et al., 2024), *Bifidobacterium adolescentis* (Zhang et al., 2022; Bustamante et al., 2022), *Bacteroides intestinalis* (Zhang et al., 2024), *Bacteroides caccae* (Mocanu et al., 2021), *Bacteroides stercoris* (Leong et al., 2020; Mocanu et al., 2021; van der Vossen et al., 2021), *Clostridium cluster* (de Groot et al., 2020) were associated with improved insulin sensitivity in MetS subjects. Additionally, responders showed notable changes in the amount of *Akkermansia muciniphila* (Mocanu et al., 2021; Kootte et al., 2017) in their feces (Leong et al., 2020; Kootte et al., 2017). *Akkermansia muciniphila, Bacteroides intestinalis*, and *Bacteroides stercoris* have also been shown to have a beneficial effect on human glucose metabolism and have been connected to T2DM (Gurung et al., 2020). *Alistipes onderdonkii* (Leong et al., 2020; Zhang et al., 2022), *Bifidobacterium bifidum* (Zhang et al., 2022), *Bifidobacterium adolescentis* (Zhang et al., 2022; Bustamante et al., 2022), *Bacteriodes vulgatus* (Zhang et al., 2022), *Christensenellaceae spp* (Mocanu et al., 2021; Zhang et al., 2024), *Roseburia spp* (Zhang et al., 2024), *Faecalibacillus intestinalis* (Zhang et al., 2024), etc. were associated with weight loss in recipients after FMT, with a decrease in the relative abundance of *E. coli* and an increase in the relative abundance of *Faecalibacterium prausnitzii, Alistipes shahii, Alistipes onderdonkii* increases were associated with improved A/G ratios (Leong et al., 2020). FMT also causes the subject's gut microbiome's *Prevotella*/*Bacteroides* ratio (P/B ratio) to rise, with Prevotella replacing Bacteroides as the dominant genus (Leong et al., 2020). This *Prevotella*-type trait has been demonstrated to be more advantageous for weight loss when combined with a high-fiber diet. The percentage of *Prevotella* was negatively correlated with insulin levels (Hjorth et al., 2018; Hjorth et al., 2019).

#### 4.1.2 Gut phages

Although gut bacteria are usually the focus of FMT research, the role played by gut viruses in FMT has been under scrutiny. Phage components make up the majority of viral components (Gregory et al., 2020), and phage therapy is becoming more and more popular as multidrug-resistant bacteria appear. In many ecosystems, phages can influence microbial populations by targeting the infection of particular bacteria and either killing them or incorporating their genes into their genomes (de Jonge et al., 2019), and it has been demonstrated that transplanting feces from which bacteria have been removed (Rasmussen et al., 2020) can effectively treat human rCDI (Ott et al., 2017).

A double-blind, randomized, placebo-controlled FMT study of 87 obese adolescents concluded that FMT promoted stable changes in recipient phage composition and increased the variability and diversity of phage and bacterial populations (Zuppi et al., 2024). Pilar et al. studied alterations in the gut phage community and phage dynamics in MetS subjects following FMT treatment. They discovered that there were notable alterations in the gut viral community following FMT treatment, with recipients' phage communities of responders and donors showing more overlap, and subjects who did not respond to treatment showing more differences between their respective viral communities and their donors (Manrique et al., 2021). Subsequently, Koen et al. conducted a small-scale fecal filtrate transplantation (FFT) and discovered that the FFT group could quickly (day 2) change the makeup of the phage viral or virus-like particle (VLP) phage group. These changes could be caused by virulence interactions that infect the donor phage, lyse the recipient bacteria, or introduce a novel donor phage, which thus induces the replication of the native phage, though this effect is temporary. By looking at the bacterial hosts of these different abundances of phages, it was found that one of the bacterial hosts is the butyrate producer *Roseburia intestinalis*, and other bacterial species are also thought to be indirectly associated with obesity and MetS (Wortelboer et al., 2023). These findings imply that phages are essential for controlling the intestinal environment. To learn more about how phages change the recipient's gut bacterial ecology and the mechanism of their effect on MetS, future research may concentrate on employing phages to target particular bacteria.

### 4.2 Metabolomics

Metabolomics is the biological science discipline that uses high-throughput techniques to detect and quantify all of the minute molecules or metabolites present in a cell, tissue, or organ (Marchesi and Ravel, 2015; Duan et al., 2025; Chen et al., 2019). Metabolomics, the study of small molecules (less than 1500 Da) present in any type of biological material, including blood, urine, feces, and tissues, helps us better understand how the host and gut microbiome interact. Gas chromatography-mass spectrometry (GC–MS), liquid chromatography-mass spectrometry (LC-MS), and capillary electrophoresis-mass spectrometry (CE-MS) are the three most widely used metabolomics analytical techniques (Chen et al., 2019). The gut microbiota and host cells interact intricately to support the body's metabolization capabilities, and the gut microbiota can influence the state of the host through the production of metabolites. Metabolites are end products or intermediates of microbial metabolism; some are directly produced from bacteria, while others are transformed from food or substrates derived from the host (Lamichhane et al., 2018). The relationship between metabolic diseases and imbalances in gut microbiota can be clarified by combining the fields of macro-genomics and metabolomics. Following the FMT intervention, there were variable degrees of changes in the amounts of metabolites assessed in both plasma and feces, with the changes mostly affecting BAs, SCFAs, AAs, and some small-molecule lipids.

#### 4.2.1 Bile acid metabolism

**Fig. 3:**
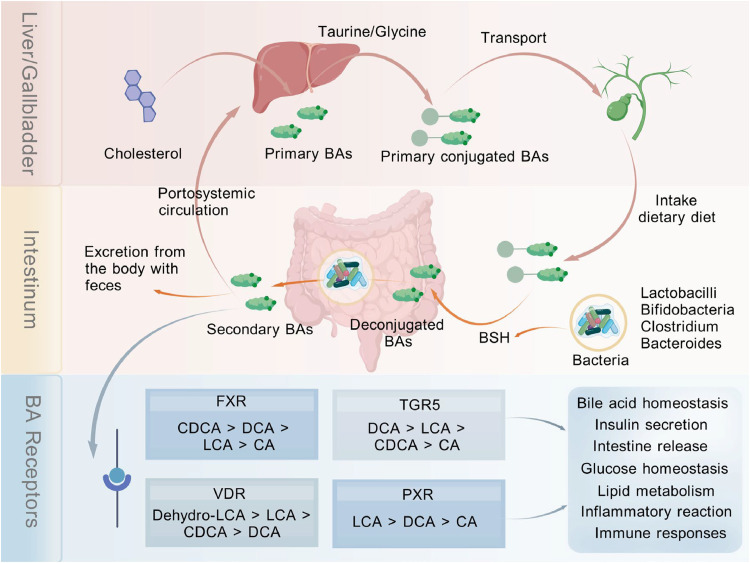
Bile acid metabolism diagram. To be more precise, cholesterol is converted into primary BAs by the liver. The primary BAs are attached to either glycine in humans or taurine in mice and then transported to the gallbladder for storage as bile. When ergonomics is consumed in a dietary diet, primary conjugated BAs in the bile are released into the intestinal lumen. Intestinal bacteria that contain functional BSH, such as Lactobacilli, Bifidobacteria, Clostridium, and Bacteroides, use BSH uncoupling to change primary conjugated BAs into secondary BAs, which are subsequently dehydrogenated (7-alpha-dehydroxylation) to produce compounds like DCA and LCA. This process mostly takes place in the lower part of the small intestine and the large intestine. The majority of secondary BAs can be returned to the liver for coupling, while a minor portion is expelled in the feces. BAs can operate as signaling molecules to regulate a variety of bioenergetic and cellular signaling pathways by binding to receptors. The primary bile acids CA and CDCA, as well as the secondary bile acids DCA and LCA, can act as ligands for signaling, however, their affinities for each receptor differ. BAs, bile acids; CA, cholic acid; CDCA, chenodeoxycholic acid; DCA, deoxycholic acid; LCA, lithocholic acid; BSH, bile salt hydrolase; FXR, farnesoid X receptor; VDR, vitamin D receptor; PXR, Pregnane X receptor; TGR5, Takeda G-protein receptor 5. Created with BioGDP.com (Jiang et al., 2024).

Bile acids (BAs) are small molecules that are converted by the liver from cholesterol (Ridlon et al., 2006; Ridlon et al., 2014). The liver and intestines are where BAs are primarily processed (Fig. 3). Primary BAs are made from cholesterol in the liver by enzyme action. They are mostly composed of cholic acid (CA) and chenodeoxycholic acid (CDCA). Prior to being released into the intestinal lumen, they are bound to taurine in mice or glycine in humans. Following their passage through the gallbladder, these primary BAs proceed with bile via the colon, where gut microbes convert them into secondary BAs such as deoxycholic acid (DCA) and lithocholic acid (LCA) (Ridlon et al., 2006; Ridlon et al., 2014; Collins et al., 2023). BAs act as signaling molecules that bind to receptors and regulate host metabolism, including insulin production, glucose homeostasis, lipid metabolism, immunological response, and inflammatory response (Collins et al., 2023; Poland and Flynn, 2021).

When compared to a placebo, it was discovered that FMT enhanced the metabolism of BAs by gut bacteria, which caused a shift in the bile acid profile towards that of the donor (Allegretti et al., 2020). The composition of the fecal metabolome's BAs, predominantly bile acid salts, was altered, and allogenic FMT significantly increased the excretion of fecal bile acid salts, even though it did not affect fasting or postprandial plasma total bile acid concentrations (Kootte et al., 2017). Furthermore, enhanced gut microbiome α-diversity was adversely correlated with baseline tauro-muricholic acid (TMCA) levels (Zhang et al., 2024). Pieter et al. performed allogenic FMT on a MetS acceptor using a MetS donor and discovered that the MetS acceptor group had higher plasma levels of taurolithocholic acid, glycolithocholic acid, and lithocholic acid while hyocholic acid levels were lower. Plasma levels of deoxycholic acid did not change, while fecal levels of deoxycholic acid, isolithocholic acid, and lithocholic acid increased significantly (de Groot et al., 2020). Simultaneously, a correlation was seen between the alterations in BA concentration and microbial levels following FMT intervention. Zhengxiao Zhang et al. discovered that the implantation of freshly transferred microbiota and the improvement of gut microbiota α-diversity were negatively connected with several BAs, including tauro-muricholic acid, glycocholic acid, glycodeoxycholic acid, and glycochenodeoxycholic acid. They also discovered that BAs may act as environmental stressors, selecting bacteria with adaptive traits necessary for long-term colonization in the gut (Zhang et al., 2024).

Currently, there are no systematic evaluation trials of the correlation between bacterial abundance and fecal or plasma BAs levels in the same samples after FMT treatment of patients with MetS (Zhang et al., 2024; Kootte et al., 2017; de Groot et al., 2020). However, Jessica et al. conducted a correlational trial in obese patients (without diabetes, MetS, or non-alcoholic fatty liver disease) (Bustamante et al., 2022). They found that FMT enrichment of *Paraprevotella, Longibaculum, Clostridium hylemonae*, and *Desulfovibrio fairfieldensis* may contribute to the effect of FMT in enhancing gut microbial bile acid metabolism and/or slowing the development of glucose intolerance. *Bifidobacterium adolescentis, Bacteroides ovatus, Faecalibacterium prausnitzi*, and *Phocaeicola dorei* may all contribute to the metabolism of intestinal bile acids. Secondly, by examining the impact of FMT on the expression of genes involved in bacterial bile acid metabolism, it was discovered that FMT only reduced *BaiB* and *BaiE* but did not impact the abundance of most known gut bacterial bile acid metabolic genes (Bustamante et al., 2022).

#### 4.2.2 Short-chain fatty acid metabolism

Short-chain fatty acids (SCFAs) are metabolic byproducts of microbial fermentation of dietary fibers. SCFAs play a critical role in maintaining intestinal epithelial barrier integrity, lipid metabolism, regulating appetite, maintaining glucose homeostasis, modulating the immune system, and reducing inflammatory responses (Morrison and Preston, 2016). Acetate, propionate, and butyrate are the three main SCFAs produced by intestinal microbiota in the large intestine when indigestible dietary fiber is fermented. There is a negative correlation between acetate levels and insulin resistance in humans (Yamaguchi et al., 2016). Propionate stimulates the satiety hormones Peptide YY (PYY) and GLP-1 to be released from the digestive tract, which results in a decrease in energy intake. The participants who took propionate supplements saw a decrease in body weight, liver fat, and abdominal adipose tissue while also maintaining insulin sensitivity (De Vadder et al., 2014). Butyrate provides energy to intestinal epithelial cells, keeps colon cells healthy, and maintains intestinal epithelial integrity (Mathewson et al., 2016). Butyrate is also a potent inhibitor of the enzyme histone deacetylase (HDAC), which regulates gene expression (Wang et al., 2018).

After FMT, fecal acetate and propionate levels increased significantly, whereas butyrate levels remained relatively unchanged (Kootte et al., 2017). Regarding the production of acetate and propionate, it has been demonstrated that *Bacteroides* and *Prevotella* are both recognized producers of these compounds from SCFAs (Chen et al., 2017; Morotomi et al., 2012). We also discovered that following FMT intervention, the abundance of *Bacteroides* and *Prevotella* in the gut increases. As for butyrate, Eduard et al. discovered that the *Intestinimonas genus* clustered with *Prevotella* in their post-FMT counts of the gut microbiome (van der Vossen et al., 2021). This *Intestinimonas genus* can produce butyrate from sugars as well as butyrate, hemolysin, and fructose lysine from acetate and lactate (Bui et al., 2016). In addition, Pieter et al. discovered a substantial correlation between responder status and an increase in the butyric acid-producing *Anaerostipes hadrus* (Zhang et al., 2022). However, we found an increase in butyrate-producing bacteria after FMT, yet fecal butyrate levels did not increase. This might be the result of the colonocytes quickly digesting the produced butyrate, leaving little for measurement. Furthermore, it was discovered that microbial assemblages dominated by *Prevotella* were able to produce more SCFAs than microbial assemblages dominated by *Bacteroides* (Chen et al., 2017).

Naturally, several studies have not discovered any discernible, noteworthy changes in SCFAs following FMT therapies. SCFAs are metabolic byproducts of the microbial fermentation of dietary fiber, dietary conditions may also have an impact on the amount of SCFAs in the feces following FMT. In both trials, the combination of other dietary interventions (fiber intervention plus FMT treatment (Mocanu et al., 2021), Mediterranean diet intervention plus FMT treatment (Koopen et al., 2021)) may have disguised the true change in the levels of fecal SCFAs after FMT.

#### 4.2.3 Amino acid metabolism

Amino acids (AAs) are important components of structural proteins such as proteins and enzymes, and are also involved in energy provision and several physiological functions (Kelly and Pearce, 2020). They are obtained through dietary intake or synthesized in the body. Common AAs include leucine, isoleucine, valine, histidine, arginine, and tryptophan. These AAs regulate protein synthesis, glucose and lipid metabolism, insulin sensitivity, hepatocyte proliferation, and are associated with human immune cell function (Kelly and Pearce, 2020), serving a crucial role in maintaining homeostasis.

Autologous FMT was predominantly associated with alterations in oxidative stress and lipid-related metabolites, whereas allogenic FMT was primarily associated with changes in AA concentrations (Kootte et al., 2017). Fen Zhang et al. discovered that an increased abundance of l‑serine and glycine biosynthesis super pathways was positively associated with weight loss in obese subjects after FMT (Zhang et al., 2022). Furthermore, the concentration of AA in the gut changes. Plasma levels of tryptophan, kynurenine (Kootte et al., 2017), N-acetyltryptophan (van der Vossen et al., 2021), gamma-aminobutyric acid, phenylalanine (Kootte et al., 2017), gamma-glutamylmethionine, and propionylglycine (van der Vossen et al., 2021) were all altered following FMT intervention.

Tryptophan (Trp) is an essential amino acid that serves as a biosynthetic precursor for a large number of metabolites (Xue et al., 2023; Su et al., 2022). Trp can be converted into a range of tryptophan metabolites via the kynurenine (Kyn), the indole, and the 5-hydroxytryptamine (HT) pathway. Most of the Trp is converted to N-formylkynurenine (NFK) via the Kyn pathway by tryptophan-2,3-dioxygenase (TDO), indoleamine-2,3-dioxygenase 1 (IDO1), and indoleamine-2,3-dioxygenase 2 (IDO2), and arylformamidase subsequently converts it to Kyn (Xue et al., 2023). Kyn metabolites have a variety of biological functions, including regulation of immune cell function and immunological and inflammatory responses (Su et al., 2022). Very small amounts of Trp are converted into indoles and their derivatives by the gut microbiota. These indole compounds promote GLP-1 secretion, which delays stomach emptying and reduces appetite (Xue et al., 2023). The gut microbiota can affect all three of Trp's metabolic pathways and yield tryptophan metabolites that are necessary for maintaining epithelial cell structure and function of pancreatic β-cells (Bansal et al., 2010) and insulin secretion (Holst, 2007). One of the metabolites produced from Trp, N-acetyltryptophan, has a strong correlation with almost all MetS characteristics, including lipid levels, insulin resistance, obesity, and blood pressure (Mirzaei et al., 2024). Moreover, the tryptophan-kynurenine pathway (Trp-Kyn) has been recognized as a potential target for the treatment of diabetes. This pathway has also been found to be intimately associated with obesity and diabetic complications (Kozieł and Urbanska, 2023). Gamma-aminobutyric acid levels were more significant in the allogenic FMT group (Kootte et al., 2017) and were positively correlated with insulin sensitivity in rodents (Tian et al., 2011). Phenylalanine, an aromatic amino acid (AAA), has been connected to obesity, T2DM, and MetS (Hernandez-Baixauli et al., 2020), while AAAs are also able to influence the gut microbiota on the 5-HT pathway (Gao et al., 2019). Gamma-glutamylmethionine, the initial byproduct of glutathione degradation, transports AAs in mammalian tissues. Increased levels of gamma-glutamylmethionine reflect decreased levels of glutathione (van der Vossen et al., 2021), which are associated with glucose metabolism and improved insulin resistance (Nguyen et al., 2013). Plasma levels of propionylglycine, a derivative of propionate, have been connected to insulin levels (van der Vossen et al., 2021) and the early stages of diabetic nephropathy (Nguyen et al., 2013). It has also been linked to DNA methylation, which corrects aberrant protein expression (Lu et al., 2018). Overall, changes in AA levels after FMT were associated with metabolic improvements in allogenic patients.

#### 4.2.4 Lipid metabolism

Lipid metabolism includes the production and degradation of lipids, such as cholesterol, phospholipids, fatty acids, triglycerides, and plasma lipoproteins (Schoeler and Caesar, 2019). Lipids have a critical role in MetS as a component of metabolomics. In addition to playing key roles in signaling, immunological responses, and the progression of sickness, they can be engaged in energy storage and the construction of cell membrane structures. The gut microbiota regulates the host's lipid metabolism (Wang et al., 2016).

Our summary analysis of plasma metabolites after FMT indicates that most are involved in lipid metabolism. In particular, 1-palmitoylglycerol, 3-hydroxystachydrine (van der Vossen et al., 2021), 4-hydroxyphenylpyruvate (de Groot et al., 2020; van der Vossen et al., 2021), and lactosyl-N-behenoyl-sphingosine were elevated in the allogenic FMT group, whereas lactosyl-N-behenoyl-sphingosine was shown to be decreasing in responders. Sphingomyelin, tricosanoyl-sphingomyelin, and 2‑hydroxy-3-methylvalerate were all markedly elevated in the autologous FMT group. Sphingomyelin and tricosanoyl-sphingomyelin alterations were less pronounced in the allogenic FMT group than in the autologous FMT group (van der Vossen et al., 2021).

Compared to the autologous FMT group, the allogenic FMT group has a significantly higher level of 1-palmitoylglycerol. This monoacylglycerol accumulates in the white and brown adipose tissue of alpha/beta-hydrolase domain-6-KO (ABHD6-KO) mice, whereas the peroxisome, which is important for the function of brown adipose peroxisome proliferator-activated receptor (PPAR) alpha and gamma are activated by it (Zhao et al., 2016). When combined with GLP-1, tranylcypromine, or alpha-ketoisocaproic acid, it also exhibits a synergistic effect on insulin secretion in pancreatic β-cell specific ABHD6-KO mice (Zhao et al., 2015). 3-hydroxystachydrine, a proline derivative, was significantly associated with lower systolic or diastolic blood pressure in people who were on the Dietary Approaches to Stop Hypertension (DASH) diet (Kim et al., 2023). 4-hydroxyphenylpyruvate has also been found to be associated with positive reactions to FMT (de Groot et al., 2020). Overall, the majority of these lipids that were significantly increased in the allogenic group showed some beneficial effects. We discovered that the autologous FMT group's plasma metabolite changes were primarily associated with sphingolipids. Sphingolipids are a class of lipids that are usually composed of a backbone of sphingomyelin bases that are modified to produce ceramides. Then, ceramides can undergo additional modification to produce more complex compounds such as sphingolipids and sphingoglycolipids (Hanamatsu et al., 2014; Merrill, 2002). Lactosyl-N-behenoyl-sphingosine, one of the ceramides in the sphingolipid family, acts as a metabolic messenger to inhibit insulin-stimulated glucose uptake, driving insulin resistance as well as raising the risk of cardiometabolic diseases (Summers et al., 2019). Ceramide has a positive correlation with both insulin resistance and fasting glucose, and interventions that lower ceramide may both lessen insulin resistance and prevent diabetes (Chaurasia and Summers, 2021). Sphingomyelin, the only non-glycerophospholipid lipid present in cell membranes, is produced by sphingolipid metabolism. Obese patients have higher plasma levels of sphingomyelin species with saturated acyl chains. These species have a favorable correlation with obesity, insulin resistance, atherogenic dyslipidemia, and liver function (Hanamatsu et al., 2014). Furthermore, sphingomyelin subsets correlate with worsened insulin sensitivity (de Groot et al., 2020). Secondly, regarding 2‑hydroxy-3-methylvalerate, it was significantly and positively correlated with insulin resistance as well as intermuscular and subcutaneous adipose tissue inflammation (Lustgarten et al., 2013). These lipids all have some detrimental effects on metabolism. In contrast, FMT did alter the plasma metabolome of MetS patients in a positive direction.

### 4.3 Epigenomics

Epigenomics is a discipline that studies the regulation of gene expression by chemical modifications without altering the DNA sequence. It primarily consists of DNA methylation, histone modification, non-coding RNA regulation, and chromatin three-dimensional structure remodeling (Allis and Jenuwein, 2016). One of them is DNA methylation, a significant epigenetic modification in which specific bases on the DNA sequence are added to methyl groups by covalent bonding in the form of DNA methyltransferase (DNMT) to change the function of genes and affect gene expression (Mattei et al., 2022; Dor and Cedar, 2018). This process does not alter the DNA sequence, but can significantly affect the transcriptional activity of a gene (Dor and Cedar, 2018).

The gut microbiota and host physiology are connected by the innate and adaptive immune systems and/or plasma metabolites (Qin and Wade, 2018; Krautkramer et al., 2016; Kumar et al., 2014; Takahashi et al., 2011). FMT has a substantial effect on the host plasma metabolome as well as the immune cell epigenome. The effects of autologous and allogenic (lean donor) FMT on the epigenomic (DNA methylation) reprogramming of peripheral blood mononuclear cells (PBMCs) from people with MetS were thus investigated further. Peripheral blood cells express most genes encoded by the human genome that respond to physical changes in the macro- and micro-environment (Liew et al., 2006). While the precise mechanisms responsible for the epigenetic changes in MetS remain unclear, studies have indicated that microbial metabolites, specifically SCFAs, could serve as co-substrates for epigenetic modifying enzymes (Krautkramer et al., 2017).

The DNA methylation of the Actin filament-associated protein 1 gene (AFAP1; cg04751533) in host PBMCs underwent considerable alteration after FMT, with the change being more pronounced in responders from the allogenic group. *Prevotella* was found to be negatively connected with the cg04751533 (CpG locus) in AFAP1 in a joint analysis with the microbiome, and reduced methylation of the cg04751533 locus in the AFAP genome was associated with diastolic blood pressure in Europeans (Kazmi et al., 2020). Trichosanoyl-sphingomyelin was the metabolite that showed the strongest correlation with AFAP1-cg04751533 (van der Vossen et al., 2021). The AFAP1 gene is associated with mitochondrial function. Although research on AFAP 1 and Actin filament-associated protein 1 antisense RNA 1 (AFAP1-AS1) has concentrated on cancer cells and tumors, Yutaro et al.'s related study discovered that increased expression of AFAP1 was associated with altered glucose metabolism in human cerebral capillary endothelial cells, and correlated with inflammation (Hoshi et al., 2017).

Furthermore, the protein kinase, DNA-activated, catalytic subunit (PRKDC; cg22338356), and histone deacetylase 4 (HDAC4; cg01114124) showed the most DNA methylation alterations in the autologous group. A co-analysis with plasma metabolites revealed that HDAC4 was associated with changes in sphingomyelin, whereas PRKDC and propionylglycine displayed a linear correlation. PRKDC genes are down-regulated in T2DM patient PBMCs (Manoel-Caetano et al., 2012), whereas HDAC genes are important in the pathogenesis of MetS and T2DM (Davegårdh et al., 2018). However, the relationship between methylation at individual sites and gene expression profiles is complex, and since many sites lack distinct gene associations, more mechanistic research is needed to dissect the relationships between loci and genes. Further investigation is also required to explore the reasons for these changes after FMT and the relationship between these changes in DNA methylation sites and MetS.

## 5 Discussion

MetS is a global health concern of the 21st century and a serious threat to human health (Cefalu et al., 2015; Neeland et al., 2024), with current medical strategies proving ineffective. MetS may develop into additional metabolic diseases or disorders if treatment is not received (Ndumele et al., 2023). Due to the ongoing lack of progress in curbing this epidemic, an increasing number of studies have begun to focus on the relationship between MetS and gut microbes, shifting attention toward novel strategies (Nicholson et al., 2012; Utzschneider et al., 2016). MetS is caused by a complicated web of causes, including extrinsic factors like diet (Maifeld et al., 2021) and lifestyle choices, and intrinsic elements like gut microbiota and genetics (Cuevas-Sierra et al., 2019). The gut microbiota may affect several MetS risk variables, and it offers both an inherent chance to affect MetS and the ability of extrinsic factors to intervene. On the other hand, FMT is essentially the transplantation of functional gut microbiota into the gastrointestinal tract of individuals with MetS via feces from healthy individuals to re-establish functional gut microbiota, which in turn influences the recipient's metabolism (Cammarota et al., 2017).

Data from clinical investigations of FMT have shown that after 6 weeks of FMT intervention, lean-donor FMT can help MetS participants lose weight (including visceral fat), improve insulin resistance and peripheral insulin sensitivity, and lower subjects' blood pressure (Mocanu et al., 2021). Single-sample allogenic FMT can produce short-term, significant, and beneficial therapeutic effects. Changes in the gut microbiota composition in MetS participants lead to changes in plasma and fecal metabolites, and these changes show great variation between patients. In addition, there was an overall lack of long-term clinical effects of FMT. When evaluated again at 18 weeks, alterations in gut microbiota and metabolites were found to return to baseline status after cessation of the FMT intervention. Week 12 following the FMT intervention was also discovered to be a potentially valuable monitoring point (Leong et al., 2020; Kootte et al., 2017).

Microbiomics investigations synthesize microbial diversity, abundance, functional activity, interactions, and relationships with the environment at baseline and after FMT intervention (Doyle et al., 2025; Duan et al., 2025). Following FMT intervention, there was a significant change in microbiota composition (beta diversity) (Mocanu et al., 2021), Shannon diversity (Leong et al., 2020; Zhang et al., 2024), and microbial abundance (Mocanu et al., 2021; Wu et al., 2023). By summarizing the microbiome study, we determined the more variable microbial species by relating the phylum, genus, and microbial species to the body weight and insulin sensitivity of MetS patients. Meanwhile, metabolomics data help us to study and identify relevant biomarkers related to complex clinical phenotypes in different biological systems to reveal molecular mechanisms of disease and to monitor disease and risk assessment (Marchesi and Ravel, 2015; Duan et al., 2025; Chen et al., 2019). Following FMT intervention, metabolites such as BAs, SCFAs, and AAs underwent some degree of alteration. Combining microbiomics and metabolomics can help to clarify the connection between metabolite disruption and gut microbiota imbalance. In terms of epigenomics, we found that FMT had a significant effect on the epigenomic reprogramming of PBMCs in patients with MetS. The DNA methylation of the AFAP1 gene in host PBMCs was altered significantly, and other genes, including PRKDC and HDAC4, were altered to a greater extent (van der Vossen et al., 2021). Epigenomics helped us to explore the mechanisms of microbial, metabolite, and clinical symptom improvement after FMT at the genetic level (Table 2).

**Table 2:** Changes in gut microbiota in metabolic syndrome or metabolic syndrome combined with obesity after FMT.

species	Post-FMT relative abundance	Post-FMT impacts	References
*Alistipes finegoldii*	Increase	(a) relative abundance may have a beneficial effect on the metabolic profile of patients; (b) relative abundance may be associated with the lack of improvement in insulin sensitivity observed in non-responders; and (c) relative abundance may be associated with an improvement in the A/G ratio.	(Leong et al., 2020; Zhang et al., 2024)
*Alistipes shahii*	Increase	(a) strong negative correlations with several bile acids, including glycine-conjugated bile acids and taurine-conjugated bile acids; (b) negative correlations with IL-6 and TNF-α; (c) a negative correlation with IL-6 and TNF-α; and (d) a possible association with improved A/G ratio.	(Leong et al., 2020; Mocanu et al., 2021) (Zhang et al., 2024)
*Alistipes onderdonkii*	Increase	(a) the strongest correlation with weight loss in post-FMT recipients; (b) a positive correlation with organic acids and their derivatives in fecal metabolites; and (c) a possible correlation with improved A/G ratios.	(Leong et al., 2020; Zhang et al., 2022)
*Akkermansia muciniphila*	Increase	(a) significant changes in *Akkermansia muciniphila* fecal abundance were observed in responders; (b) were associated with improvements in insulin sensitivity and reductions in total plasma cholesterol; and (c) were negatively correlated with body weight and BMI.	(Mocanu et al., 2021; Kootte et al., 2017; Depommier et al., 2019; Rodrigues et al., 2022; Dao et al., 2016)
*Bifidobacterium bifidum*	Increase	(a) relative abundance was associated with weight loss in obese subjects; (b) relative abundance was significantly and positively correlated with levels of metabolites related to amino acid metabolism (hexanoylglycine, l-malate, l-homocitrulline, and N6-acetyl-l-lysine) and eicosapentaenoic acid.	(Zhang et al., 2022)
*Bifidobacterium adolescentis*	Increase	(a) relative abundance was associated with weight loss in obese subjects; (b) relative abundance showed a significant negative correlation with HOMA-IR, an indicator of urgency in the treatment of T2DM; and (c) relative abundance was positively correlated with the production of deoxycholic acid (especially the glycine-bound form), which may play a role in the conversion of primary bile acids to secondary bile acids.	(Zhang et al., 2022; Bustamante et al., 2022)
*Bifidobacterium pseudocatenulatum*	Increase	(a) a potential probiotic that produces acetic acid from dietary carbohydrates, which may be associated with significantly higher levels of acetate in feces after FMT; and (b) may have been obtained from the donor group.	(Kootte et al., 2017; Koopen et al., 2021)
*Bacteroides dorei*	Increase	Increased differences in the abundance of this species were observed in both the donor and allogenic groups, possibly as a result of the implantation of a bacterial strain from a healthy donor source.	(Koopen et al., 2021)
*Bacteroides fragilis*	Increase	(a) *Bacteroides fragilis* abundance was negatively correlated with fat and animal protein intake; (b) positively correlated with carbohydrate intake; and (c) negatively correlated with the baseline pro-inflammatory biomarkers CRP and TNF-α.	(Zhang et al., 2024)
*Bacteroides ovatus*	Increase	(a) positively correlates with unconjugated goose deoxycholic acid; (b) may have a potential role in bile acid uncoupling; and (c) changes in levels are likely to correlate with improved A/G ratios.	(Leong et al., 2020; Bustamante et al., 2022)
*Bacteriodes vulgatus*	Increase	(a) correlation with weight loss in subjects after FMT; (b) an increase in the phosphopantothenate biosynthesis I pathway in receptors with weight loss after FMT, suggesting that this function may be one of the potential mechanisms to help *Bacteriodes vulgatus* combat obesity; and (c) abundance of *Bacteriodes vulgatus* was significantly and positively correlated with amino acid metabolism-related metabolites and eicosapentaenoic acid were significantly positively correlated.	(Zhang et al., 2022)
*Bacteroides intestinalis*	Decrease(non-responders)	(a) may have beneficial effects on the metabolic profile of patients; and (b) may be associated with the lack of improvement in insulin sensitivity observed in non-responders.	(Zhang et al., 2024)
*Bacteroides caccae*	Increase	Significantly correlated with HOMA2-IR and insulin sensitivity.	(Mocanu et al., 2021)
*Bacteroides stercoris*	Increase	(a) significantly correlated with HOMA2-IR and insulin sensitivity; and (b) acted as a dominant strain replacing the dominant strain in the receptor.	(Leong et al., 2020; Mocanu et al., 2021) (van der Vossen et al., 2021)
*Clostridium symbiosum*	Increase	(a) may be involved in metabolic regulation through the catabolic production of secondary bile acids; and (b) increased numbers of intestinal *Clostridium symbiosum* may lead to increased GLP-1 secretion.	(Kootte et al., 2017)
*Clostridium cluster*	Increase	Positively correlated with changes in peripheral insulin sensitivity.	(de Groot et al., 2020)
*Christensenellaceae* spp.	Increase	(a) *Christensenellaceae* spp. abundance was negatively correlated with obesity; and (b) was associated with metabolic health as well as reduced visceral adipose tissue and BMI.	(Mocanu et al., 2021; Zhang et al., 2024)
*Desulfovibrio piger*	Decrease	(a) reduced abundance in donors as well as receptors; and (b) increased *Desulfovibrio* spp. may be associated with metabolic deterioration in metabolic syndrome receptors.	(Koopen et al., 2021; de Groot et al., 2020)
*Faecalibacterium prausnitzii*	Increase	(a) may be associated with improved A/G; (b) is positively correlated with lithocholic acid and may play a role in the conversion of primary to secondary bile acids.	(Leong et al., 2020; Bustamante et al., 2022)
*Faecalibacillus intestinalis*	Increase	(a) a negative correlation with obesity; and (b) an indication that donor bacteria are able to colonize and coexist with the recipient microbiota in responders (donor-to-recipient transplantation of the gut microbiota).	(Zhang et al., 2024)
*Prevotella copri*	Increase	(a) an efficient producer of BCAAs; (b) the percentage of *Prevotella* after treatment is negatively correlated with insulin levels; and (c) *Prevotella copri* is consistently translocated and stably colonized in FMT receptors.	(van der Vossen et al., 2021)
*Roseburia* spp.	Increase	(a) a negative correlation with obesity; and (b) an indication that donor bacteria are able to colonize and coexist with the recipient microbiota in responders.	(Zhang et al., 2024)
*Ruminococcus callidus*	Decrease(non-responders)	(a) may have beneficial effects on the metabolic profile of patients; and (b) may be associated with the lack of improvement in insulin sensitivity observed in non-responders.	(Zhang et al., 2024)
*Ruminococcus torques*	Decrease	(a) low abundance in responders' baseline fecal samples; and (b) possible association with metabolic deterioration.	(Mocanu et al., 2021; Kootte et al., 2017)

A/G, android-to-gynoid-fat; BCAA, branched-chain amino acids; HOMA-IR, Homeostasis Model Assessment-nnInsulin Resistance; FMT, fecal microbial transplantation; GLP-1, glucagon-like peptide 1.

Integration and analysis of multi-omics models is an important component in bioinformatics and data science. The joint analysis between DNA methylation sites, gut microbiota, metabolites, and relevant clinical parameters allows us to understand the impact of FMT on microbes, metabolites, gene expression, and clinical symptom improvement. However, we suggest that we need further multi-omics integration approaches, biomarker selection frameworks, and machine learning/artificial intelligence studies to explore the relationship between microbes, metabolites, epigenetic modifications, and clinical parameters after FMT. Univariate testing underestimates the relevance of biological data, thereby increasing the number of false-negative errors. Therefore, to mitigate this problem and to understand how data domains from different histologies are jointly affected, further research could invoke frameworks designed for unsupervised integration of heterogeneous data. Examples include Manifold Mixing for Stacked Regularization (MMSR) (Pereira et al., 2022), a specific model-based strategy, and Multi-Omics Factor Analysis v2 (MOFA+) (Argelaguet et al., 2020), a model extension. MMSR is based on the manifold assumption, the idea that complex high-dimensional data lie on or close to a lower-dimensional manifold, and each dataset manifold is used to mix information from various modalities, and then the transformed data is fed to a stacked model which optimizes its parameters by Bayesian optimization of the joint (Pereira et al., 2022). MOFA+ inputs are multiple datasets, and this model develops a stochastic variational inference framework suitable for GPU computation, capable of analyzing datasets with potentially millions of units, as well as combining a priori for flexible structural regularization to enable joint modeling of multiple groups and data patterns (Argelaguet et al., 2020).

At the same time, supervised methods for interpreting molecular patterns across biological domains or characterizing known phenotypes are equally useful. There are also advantages of supervised multiblock methods over existing unsupervised synthesis methods. We recommend Data Integration Analysis for Biomarker discovery using Latent cOmponents (DIABLO), a multi-omics approach (Singh et al., 2019). This approach allows for data integration and characterization of biomarkers and molecular signatures, allowing for the simultaneous identification of key histological variables and differentiation of phenotypic groups during the integration process. DIABLO aims to identify coherent patterns of variation between datasets based on different phenotypes, which can be used to obtain robust biomarkers for the prediction of new samples and ultimately to improve our understanding of the molecular mechanisms that drive disease (Singh et al., 2019). Of course, DIABLO assumes that there is a linear relationship between selected histologic features to explain the phenotypic response. This assumption may not be applicable to some areas of research. In machine learning/artificial intelligence, model transparency and feature correlation are important aspects in discovering new potential treatments or research directions. In medicine, revealing the mechanisms behind a disease is often more important than the diagnosis itself. One of the most popular methods for interpreting the global predictions of a model is the importance of replacement, as a benchmark to measure the performance of the replacement data. However, this and other related methods underestimate the importance of features in the presence of covariates that cover part of the information provided by the features. To address this issue, Covered Information Disentanglement (CID) (Pereira et al., 2022), a framework that takes into account the overlap of all feature information, can be used to correct the values provided by ranked importance. CID is able to provide a truly model-independent framework for feature importance while retaining the intuitive nature of ranked importance.

The dramatic increase in data generation requires new methods capable of handling information that is inherently high-dimensional, heterogeneous, and incomplete. High-dimensional multi-omics can better cope with this problem, and the application of high-dimensional statistics to omics data helps us to perform feature selection and robust inference of biomarkers (Vinga, 2021). Meanwhile, high-dimensional multi-omics requires sparse regularization to recover robust features. Among them are Elastic Net and Group Lasso, which utilize sparsity to identify relevant feature sets and improve the interpretability of omic biomarker discovery. Lasso is a regularization technique. With lasso you can reduce the number of predictors in a regression model, identify important predictors, and select among redundant predictors. Elastic Net is a hybrid of ridge regression and Lasso regularization. Similar to Lasso, Elastic Net can generate simplified models by generating zero-valued coefficients. Empirical studies have shown that the Elastic Net technique can outperform Lasso for data with highly correlated predictor variables (Hastie et al., 2015).

Finally, in terms of the safety of the test, it is theoretically possible to spread harmful microbiota traits (DeFilipp et al., 2019). We must admit that it is difficult to determine the exact mechanism of the therapeutic effect of manipulating the microbiome via FMT because it transfers not just bacteria and viruses but also the entire microbiome along with donor human proteins and/or cells. Nonetheless, as far as clinical studies are concerned, the FMT technique seems to be generally safe. Most of the adverse events observed in trials appear to stem from initial disease severity or route of administration rather than from FMT itself. FMT was safe and well tolerated. Recipients of FMT reported only mild gastrointestinal adverse effects, including bloating, flatulence, hiccups, cramping, abdominal discomfort, irregular bowel movements, and vomiting (Aron-Wisnewsky et al., 2019). The administration of FMT can be done through oral capsules, the upper gastrointestinal route, or the lower gastrointestinal route (Nicco et al., 2020). There have been two deaths in FMT studies that may have been potentially related deaths reported: one patient died from aspiration while under anesthesia for FMT via colonoscopy, and another patient with severe CDI passed away from peritonitis, which could have been caused by the infusion method (Rossen et al., 2015). Compared to colonoscopy and nasoduodenal tube, oral capsule administration of FMT has better tolerance after the procedure. The administration of oral capsules seems to be a safer method, which could make FMT treatment more feasible and acceptable (Leong et al., 2020; Zuppi et al., 2024; Mocanu et al., 2021; Zhang et al., 2024; Zhang et al., 2022). However, we believe that it is of utmost importance to identify the relevant strains by FMT tests and multi-omics techniques, and definitive consortium of purified, clonal bacterial strains have been shown to have better results (Menon et al., 2025).

## 6 Conclusion

FMT aims to restore optimal microbial diversity and thus improve the clinical symptoms of MetS patients. However, the targeted therapeutic properties of FMT are weak at present, while they have inherently variable quality attributes and a risk of pathogen transfer. So our future questions will be based on microbiota-targeted therapy or FFT and FVT therapies specifically targeting bacteria. FMT allows us to explore microbial species, including phages and bacteria, that are advantageous for MetS therapy. Moreover, dietary factors may influence the results of FMT; therefore, systematic dietary counseling to monitor food intake before the intervention and routine dietitian monitoring and follow-up following the intervention should be considered. Furthermore, almost all current studies have demonstrated that FMT can only temporarily change insulin sensitivity and the gut microbiota, which may be another problem we must address.

FMT simultaneously affected plasma or fecal metabolite levels, gut microbial species, abundance, gene abundance, and DNA methylation of host PBMCs. Nevertheless, we discovered that studies on multi-omics are still relatively few in comparison and can provide limited therapeutic clues. Utilizing multi-omics techniques to find beneficial bacteria, providing relevant information to identify key molecules and genetic changes involved in biological effects, and understanding active metabolic pathways and mechanisms of action can provide better clues for FMT treatment of MetS. Comprehensive multi-omics analyses, as well as FMT assays, help us to identify promising microbial and metabolic cues that provide ideas for the selection of definitive consortia consisting of purified clonal bacterial strains or live biotherapeutic products, or that can be translated into phage therapy targeting specific bacteria. Indeed, even replacing the FMT program and ultimately using the gut microbiota as a therapeutic target.

## Funding

The authors reported there is no funding associated with the work featured in this article.

## CRediT authorship contribution statement

**Hanrui Wang:** Writing – original draft, Writing – review & editing, Visualization. **Jiaxing Tian:** Writing – review & editing, Supervision, Conceptualization. **Jia Mi:** Writing – review & editing, Supervision, Conceptualization.

## Declaration of competing interest

The authors declare that they have no known competing financial interests or personal relationships that could have appeared to influence the work reported in this paper.

## Data Availability

No data was used for the research described in the article.
